# Precise Identification of Glioblastoma Micro‐Infiltration at Cellular Resolution by Raman Spectroscopy

**DOI:** 10.1002/advs.202401014

**Published:** 2024-07-31

**Authors:** Lijun Zhu, Jianrui Li, Jing Pan, Nan Wu, Qing Xu, Qing‐Qing Zhou, Qiang Wang, Dong Han, Ziyang Wang, Qiang Xu, Xiaoxue Liu, Jingxing Guo, Jiandong Wang, Zhiqiang Zhang, Yiqing Wang, Huiming Cai, Yingjia Li, Hao Pan, Longjiang Zhang, Xiaoyuan Chen, Guangming Lu

**Affiliations:** ^1^ Department of Radiology, Jinling Hospital, The First School of Clinical Medicine Southern Medical University 305 Zhongshan Road East, Xuanwu Nanjing 210002 China; ^2^ Department of Medicine Ultrasonics Nanfang Hospital Southern Medical University Guangzhou 510515 China; ^3^ Department of Radiology, Jinling Hospital, Affiliated Hospital of Medical School Nanjing University 305 Zhongshan Road East, Xuanwu Nanjing 210002 China; ^4^ Department of Pathology, Jinling Hospital, Affiliated Hospital of Medical School Nanjing University Nanjing 210002 China; ^5^ Department of Neurosurgery, Jinling Hospital, Affiliated Hospital of Medical School Nanjing University 305 Zhongshan Road East, Xuanwu Nanjing 210002 China; ^6^ Department of Biomedical Engineering, College of Engineering and Applied Sciences, State Key Laboratory of Analytical Chemistry for Life Science Nanjing University Nanjing 210002 China; ^7^ Department of Radiology Nanjing First Hospital Nanjing Medical University Nanjing 210002 China; ^8^ School of Chemistry Chemical Engineering and Life Sciences Wuhan University of Technology Wuhan 430000 China; ^9^ Departments of Diagnostic Radiology, Surgery, Chemical and Biomolecular Engineering, and Biomedical Engineering, Yong Loo Lin School of Medicine and College of Design and Engineering National University of Singapore Singapore 119074 Singapore; ^10^ Clinical Imaging Research Centre Centre for Translational Medicine Yong Loo Lin School of Medicine National University of Singapore Singapore 117599 Singapore; ^11^ Nanomedicine Translational Research Program, Yong Loo Lin School of Medicine National University of Singapore Singapore 117597 Singapore; ^12^ Theranostics Center of Excellence (TCE), Yong Loo Lin School of Medicine National University of Singapore 11 Biopolis Way Helios 138667 Singapore; ^13^ Institute of Molecular and Cell Biology, Agency for Science, Technology, and Research (A*STAR) 61 Biopolis Drive, Proteos Singapore 138673 Singapore; ^14^ State Key Laboratory of Analytical Chemistry for Life Science School of Chemistry and Chemical Engineering Nanjing University Nanjing 210002 China

**Keywords:** glioblastoma, identification, micro‐infiltration, Raman spectroscopy

## Abstract

Precise identification of glioblastoma (GBM) microinfiltration, which is essential for achieving complete resection, remains an enormous challenge in clinical practice. Here, the study demonstrates that Raman spectroscopy effectively identifies GBM microinfiltration with cellular resolution in clinical specimens. The spectral differences between infiltrative lesions and normal brain tissues are attributed to phospholipids, nucleic acids, amino acids, and unsaturated fatty acids. These biochemical metabolites identified by Raman spectroscopy are further confirmed by spatial metabolomics. Based on differential spectra, Raman imaging resolves important morphological information relevant to GBM lesions in a label‐free manner. The area under the receiver operating characteristic curve (AUC) for Raman spectroscopy combined with machine learning in detecting infiltrative lesions exceeds 95%. Most importantly, the cancer cell threshold identified by Raman spectroscopy is as low as 3 human GBM cells per 0.01 mm^2^. Raman spectroscopy enables the detection of previously undetectable diffusely infiltrative cancer cells, which holds potential value in guiding complete tumor resection in GBM patients.

## Graphical abstract

This study investigates the cancer density threshold of glioblastoma infiltrating lesions identified by Raman spectroscopy. Raman spectroscopy reveals the metabolic characteristics of the infiltrating lesions and distinguishes micro‐infiltrative lesions from normal brain tissues. This demonstrates the potential of Raman spectroscopy in identifying micro‐infiltrative lesions at a cellular resolution (the figure is created with BioRender.com).

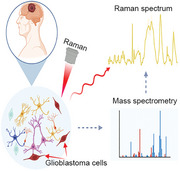

## 1 Introduction

Glioblastoma (GBM) is the most devastating brain tumor due to its highly infiltrative nature.^[^ ^1^ ^]^ Despite the use of multimodal treatments, the median survival of patients with GBMs is <1 year.^[^ ^2^ ^]^ Surgery is the cornerstone treatment for GBM and the extent of resection determines the prognosis.^[^ ^3^ ^]^ According to a previous study,^[^ ^4^ ^]^ even if the postoperative residual volume is <2.3 cm^3^, GBM recurs due to the failed removal of micro‐infiltrative lesions. Therefore, precise identification of micro‐infiltrative tumors is essential to achieving maximal safe resection and improving GBM patient survival. Current techniques used to detect bulk tumors and infiltrating lesions include magnetic resonance imaging (MRI),^[^ ^5^ ^]^ positron emission tomography (PET),^[^ ^6^ ^]^ and fluorescence imaging,^[^ ^7^ ^]^ but all of these techniques fail to identify micro‐infiltration of GBM due to their limited resolution. Therefore, to achieve maximal safe resection, strategies with high resolution to detect GBM micro‐infiltration are desperately needed.

Raman spectroscopy (RS), with high resolution,^[^ ^8^ ^]^ serves as a promising method to identify GBM micro‐infiltration. RS is a label‐free technique based on inelastic light scattering and probes molecular vibrations associated with chemical bonds in tissues.^[^ ^9^ ^]^ All constituents of tissues contribute to the Raman bands^[^ ^8^ ^]^ and subtle constituent changes can be captured by RS,^[^ ^10^ ^]^ making it highly sensitive.^[^ ^11^ ^]^ GBM micro‐infiltration can induce changes in the Raman spectra, produced as a result of alteration in the metabolites of the micro‐infiltrative lesions. Hence, RS has a great potential to identify micro‐infiltration of GBM. Nowadays, RS has been used in GBM detection,^[^ ^12^ ^]^ while little attention has been paid to identifying GBM micro‐infiltration.

Herein, we utilized RS combined with machine learning to effectively identify GBM micro‐infiltration. The GBM cell density threshold in infiltrating lesions identified by RS was as low as 3 GBM cells per 0.01 mm^2^ for the first time. Spatial metabolomics was applied to validate metabolic changes identified by RS. Additionally, we leveraged Raman imaging to visualize the different tissue types at micrometric resolution. To sum up, the current study revealed the potential of RS to identify micro‐infiltrative lesions at unprecedented resolution, which provides a novel auxiliary tool for the complete resection of GBMs in the future.

## 2 Results

### 2.1 Comparison of Raman Spectra of Normal Tissues and Infiltrative Lesions

Considering the tumor heterogeneity and variance, the murine GBM cell line, GL261, and the human GBM cell line, LN229, were included in this study. Both cell lines formed infiltrative orthotopic GBMs, migrating diffusely from the primary tumor and invading the adjacent normal regions (Figure S1, Supporting Information).

**Figure 1:**
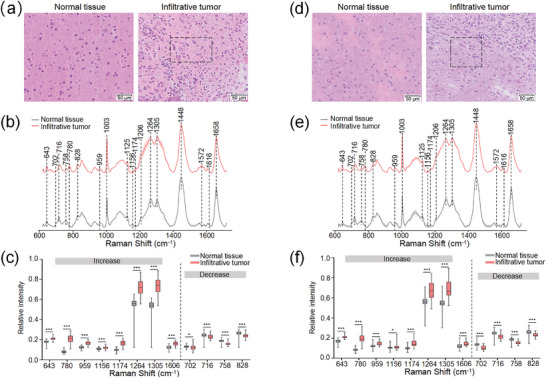
Comparison of normal brain tissues and infiltrating lesions in (a‐c) GL261 and (d‐f) LN229 tumor‐bearing mice (n = 16 per animal model). a,d) The H&E images of normal brain tissues and infiltrative tumors. The black boxes are the infiltrative areas. b,e) The average Raman spectra of normal brain tissues and infiltrative tumors (n = 64 per group). The shaded areas represent the standard deviations. c,f) Raman peaks that either increase or decrease in infiltrative tumors relative to normal brain tissues (n = 64 per group). The Raman peak intensities of 959, 1156, 1174, 1606, and 702 cm^−1^ in GL261 tumor‐bearing mice (c) were analyzed using the unpaired t‐tests, and other Raman peak intensities (c,f) were analyzed by the Mann‐Whitney test, *P < 0.05, **P < 0.01, ***P < 0.001.

To compare the spectral differences between normal brain tissues and infiltrative lesions, we collected spectra from 128 sites in GL261 and LN229 tumor‐bearing mice respectively, with each site referring to a Raman scan area of 100 × 100 µm. The hematoxylin and eosin (H&E) images showed normal tissues and infiltrative tumors, and several GBM cells infiltrated normal brain tissues in the peritumoral infiltration areas (**Figure** **1**a,d). The majority of the peaks attributed to proteins (1125 and 1156 cm^−1^), amino acids (643, 758, 1206, and 1616 cm^−1^), lipids (702, 716, 1264, 1305, and 1448 cm^−1^), and nucleic acids (780 and 1572 cm^−1^) were prominent in both normal brain tissues and infiltrative lesions (Figure 1b,e). Furthermore, we quantitatively compared the spectra intensities between infiltration lesions and normal brain tissues by statistical analysis. The differences in specific Raman peaks are presented in Figure 1c,f. The peak at 643 cm^−1^ associated with proline, a key component of collagen, was higher in the infiltration lesions compared to normal brain tissues. In contrast, the content of phospholipids in infiltrative tumors was reduced according to the 716 cm^−1^ peak. Similarly, the 758 cm^−1^ peak is attributed to tryptophan and the intensity of 758 cm^−1^ peak decreased in infiltrative lesions. The peak at 780 cm^−1^ is assigned to nucleic acids and the high peak in the infiltrative lesions reflects the high proliferation rate and enhanced nuclear mitosis of cancer. Proteins, saturated fatty acids (SFAs), and unsaturated fatty acids (UFAs)‐related peaks, such as 1156, 1174, and 1264 cm^−1^, respectively, were higher in the infiltrating lesions compared to normal tissues. The positions and major assignments of the Raman peaks are listed in the Supporting Information (Table S1, Supporting Information) according to the existing literature.^[^ ^13^ ^]^ These findings specifically illustrated that key biochemical metabolites play important roles in GBM growth and infiltration^[^ ^14^ ^]^ and the differentials in content provide a basis for RS to identify infiltrative cancer.

Several studies have reported the identification of bulk tumors using Raman techniques.^[^ ^15^ ^]^ In this study, the spectra of dense tumors were acquired from 64 sites in GL261 and LN229 tumor‐bearing mice respectively. Raman peaks assigned to the amino acids (667, 1003, and 1206 cm^−1^), lipids (1174, 1264, and 1305 cm^−1^), and nucleic acids (780 and 1572 cm^−1^) are highlighted in bulk tumors (Figure S2, Supporting Information). RS combined with machine learning effectively identified the bulk tumors with area under the receiver operating characteristic curves (AUCs) of 100% in human samples (Table S2, Supporting Information).

### 2.2 Metabolite Validation using the Spatial Metabolomics Technique

**Figure 2:**
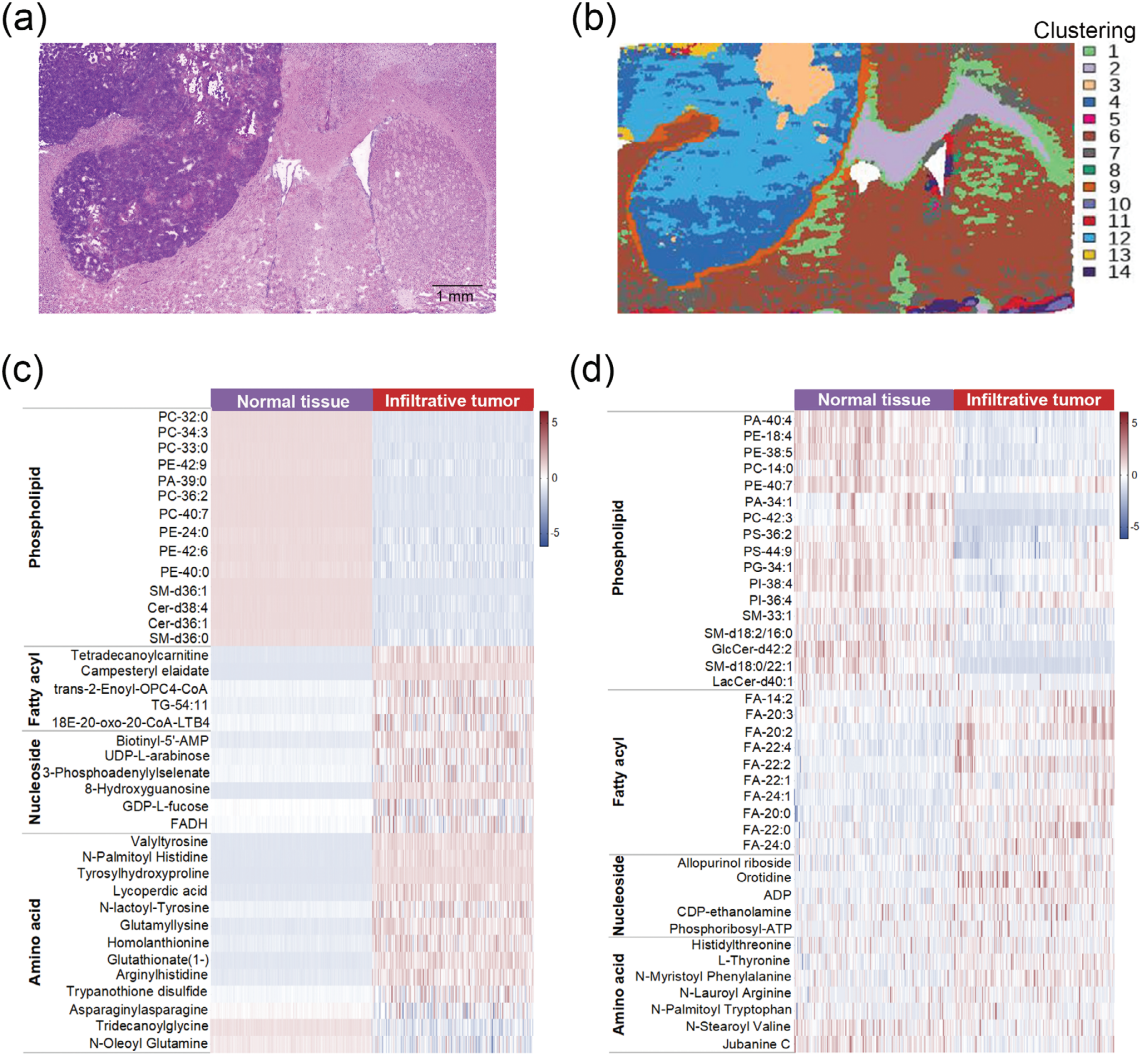
AFADESI‐MSI analysis of brain slices. H&E a) and corresponding spatial clustering images b) of different regions in the brain slices. The 1^st^, 2^nd^, 6^th^, and 7^th^ clusters were normal brains and the 9^th^ cluster was a tumor infiltration area. Heat maps of AFADESI‐MSI data in ESI+ c) and ESI− modes d) showing various classes of metabolites for normal tissues (the 1^st^, 2^nd^, 6^th^, and 7^th^ clusters) and infiltrative tumors (the 9^th^ cluster). Abbreviations: PC (phosphatidylcholine), PE (phosphatidylethanolamine), PA (phosphatidic acid), PS (phosphatidylserine), PG (phosphatidylglycerol), PI (phosphatidylinositol), SM (sphingomyelin), and Cer (ceramide).

To further confirm the biochemical metabolites observed in RS, the air flow‐assisted desorption electrospray ionization mass spectrometry imaging (AFADESI‐MSI)‐mediated spatial metabolomics technique was used. Detailed data on AFADESI‐MSI are described in the Supplemental Text (Supporting Information). **Figure** **2**a illustrates the typical H&E images of a tumor‐bearing brain slice and suggests that in addition to dense tumors, there are peritumoral infiltration areas, necrotic tissues, and different sub‐brain regions. The regions with similar metabolites were clustered and given a specific color in the spatial clustering diagram that was divided into 14 clusters (Figure 2b). According to the H&E image, the 9^th^ cluster was the tumor infiltration area and the 1^st^, 2^nd^, 6^th^, and 7^th^ clusters were normal brain tissues, which were the main areas of our study. Additionally, the 3^rd^ and 13^th^ clusters represented necrotic tissues within the tumor, and the 4^th^ and 12^th^ clusters were tumor masses. The 5^th^, 8^th^, 10^th^, 11^th^, and 14^th^ clusters were derived from the margins of the brain ventricle and slice. As shown in Figure 2c,d, the heat maps show significant differences in metabolites between infiltrative tumors (the 9^th^ cluster) and normal tissues (the 1^st^, 2^nd^, 6^th^, and 7^th^ clusters). Representative metabolites were classified into four main categories: phospholipids; fatty acyls; nucleosides; and amino acids. Compared with normal brain tissues, tumor infiltration lesions contained higher levels of fatty acyls, nucleosides, and amino acids, and lower levels of phospholipids. In addition, the metabolic profile of the tumor masses (the 4^th^ and 12^th^ clusters) was described in the Supporting Information (Figure S3, Supporting Information).

**Figure 3:**
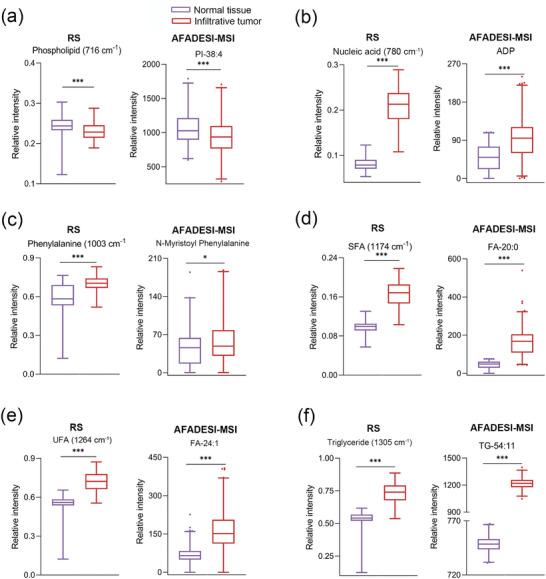
Analysis of the metabolites trend identified by RS and AFADESI‐MSI (n = 64). Selective biochemical metabolites identified by RS including phospholipids (716 cm^−1^) a), nucleic acids (780 cm^−1^) b), phenylalanine (1003 cm^−1^) c), SFAs (1174 cm^−1^) d), UFAs (1264 cm^−1^) e), triglycerides (1305 cm^−1^) f) and equivalent representative metabolites of AFADESI‐MSI. SFAs (1174 cm^−1^) (d) and TG‐54:11 (f) were analyzed using the unpaired t‐tests, and other data (a‐f) were analyzed by the Mann‐Whitney test, *P < 0.05, **P < 0.01, ***P < 0.001.

We further examined the concordance between metabolite changes detected by AFADESI‐MSI and those identified by RS. The 716 cm^−1^ peak is attributed to phospholipids. The equivalent phospholipid metabolite detected by AFADESI‐MSI was phosphatidylinositol‐38:4 (PI‐38:4). The change in phospholipids content among infiltrative lesions and normal brain tissues was consistent with the PI‐38:4 content (**Figure** **3**a). Other metabolites identified by RS, including nucleic acids, phenylalanine, SFAs, UFAs, triglycerides, and equivalent AFADESI‐MSI metabolites (ADP, N‐myristoyl phenylalanine, FA‐20:0, FA‐24:1, triglyceride‐54:11 [TG‐54:11]), are shown in Figure 3b‐f. The results demonstrated that the metabolic changes detected by RS and AFADESI‐MSI were largely congruent, thereby confirming the validity of Raman spectra‐profiled metabolic trends.

### 2.3 Raman Mapping of GBM

**Figure 4:**
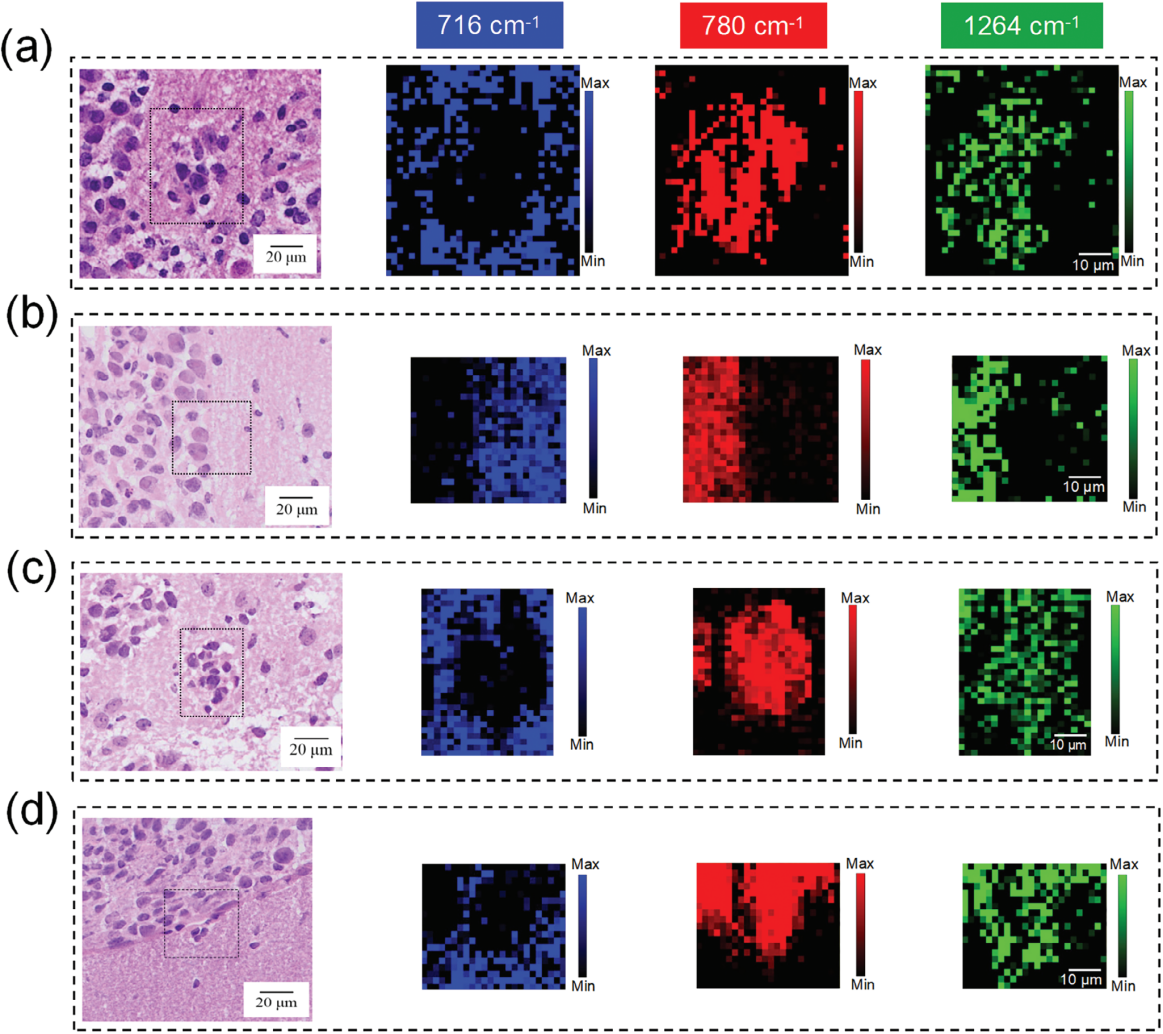
Raman imaging of GBM in GL261 a,b) and LN229 c,d) tumors. Raman imaging for infiltrative tumors (a,c) and clear tumor boundaries (b,d). The black boxes in H&E images are the approximate Raman imaging areas. Raman images visualized lesions based on the 716 (color‐coded in blue), 780 (color‐coded in red), and 1264 cm^−1^ peaks (color‐coded in green).

RS not only distinguishes infiltrative lesions based on spectra that offer biomolecular information but also visualizes lesions. We focused on the identification of infiltrative tumors and the spatial distribution of pathological tissues based on Raman imaging. The H&E and corresponding Raman images in infiltrating lesions are shown in **Figure** **4**a,c. Raman images of extracted peak amplitude maps at 716, 780, and 1264 cm^−1^ are associated with phospholipids (color‐coded in blue), nucleic acids (color‐coded in red), and UFAs (color‐coded in green), respectively. The higher the brightness of the color pixel in the Raman images, the greater the biological molecule content. Raman maps based on the 716 cm^−1^ peak exhibited significant darkness in the lesion areas but brightness in normal tissues, which suggests a lower phospholipid concentration in the infiltrating lesions compared to normal tissues. Conversely, Raman maps based on nucleic acids (780 cm^−1^) and UFAs (1264 cm^−1^) had bright pixels in the infiltrating lesions and easily visualized the lesion. Additionally, Figure 4b,d illustrates Raman maps with clear tumor boundaries, indicating that the tumor and normal tissues can be visualized using various pseudocolors. Raman images in the region of characteristic vibrations can delineate cancer outlines because tumor tissues are rich in nucleic acids and UFAs but with less phospholipids. The Raman images provided the morphology of the lesions, which matched well with H&E images. The Raman technique reveals valuable information on the detailed biochemical composition and the morphological structures of tissues in a non‐destructive and label‐free manner.

### 2.4 Correlation between the Degree of Tumor Infiltration and Raman

**Figure 5:**
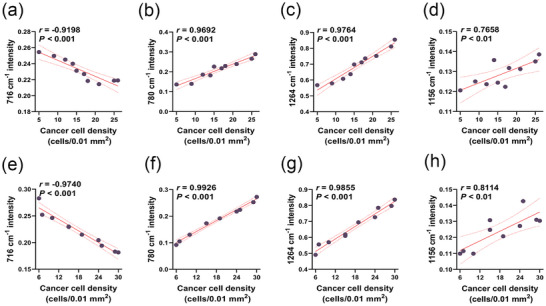
The correlation between tumor cell density and Raman intensity in a‐d) GL261 and e‐h) LN229 infiltrative lesions (n = 10). Selective Raman peaks including 716 cm^−1^ (phospholipids) (a,e), 780 cm^−1^ (nucleic acids) (b,f), 1264 cm^−1^ (UFAs) (c,g), 1156 cm^−1^ (proteins) (d,h). *r* represents the correlation coefficient. The cancer cell density = number of cancer cells/0.01 mm^2^. *P < 0.05, **P < 0.01, ***P < 0.001.

To determine the relationship between tumor cell density and the intensity of the Raman peak, a Pearson correlation analysis was performed. We analyzed four Raman peaks, including 716 cm^−1^ (phospholipids), 780 cm^−1^ (nucleic acids), 1264 cm^−1^ (UFAs), and 1156 cm^−1^ (proteins). Cancer cells were counted in the areas of 0.01 mm^2^, and the cell density was calculated. A strong negative correlation was detected between the intensity of the 716 cm^−1^ peak and the tumor cell density. The correlation coefficients were −0.9198 and −0.9740 in the GL261 and LN229 infiltrative lesions, respectively (**Figure** **5**a,e). Conversely, the intensity of the 780 cm^−1^ peak was highly positively correlated with the tumor cell density (*r* = 0.9692 and 0.9926 in the GL261 and LN229 infiltrative lesions, respectively; Figure 5b,f). Similarly, the 1264 cm^−1^ intensity had a significant positive correlation with tumor cell density and the correlation coefficients were 0.9764 and 0.9855 in the GL261 and LN229 infiltrative lesions, respectively (Figure 5c,g). In addition, a moderately positive correlation was observed between the intensity of the 1156 cm^−1^ peak and tumor cell density (Figure 5d,h), with correlation coefficients of 0.7658 and 0.8114 in the GL261 and LN229 infiltrative lesions, respectively. The peak intensity was linearly related to the metabolite content that could change as the tumor infiltration progresses.^[^ ^16^ ^]^ Our results demonstrated that the cancer cell density of the infiltrative lesions can be estimated by the spectral intensity.

### 2.5 Identification of GBM Micro‐Infiltration in Clinical Specimens

**Figure 6:**
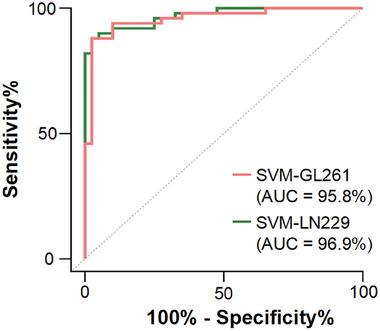
Receiver operating characteristic (ROC) curves and corresponding AUC values of SVM‐GL261 a) and SVM‐LN229 b) in human specimens.

To identify infiltrative tumors, a support vector machine (SVM), which is well‐suited for high‐dimensional Raman data, was used. 10 Raman features were selected using Pearson feature correlation analysis and ANOVA test for SVM classifier construction and are shown in Table S3 (Supporting Information). Raman features of training sets from GL261 and LN229 tumor‐bearing mice were used to develop the classification models, SVM‐GL261 and SVM‐LN229, respectively. The test sets comprising GL261 and LN229 tumor‐bearing mice that underwent contrast‐enhanced T1‐weighted image (CE‐T1WI) were utilized to validate the efficacy of the classification model. The machine learning metrics we used included classification accuracy, AUC, sensitivity, and specificity. RS combined with SVM had excellent performance in accurately identifying infiltrative tumors, and the AUCs were 98.4% and 96.1% in GL261 and LN229 tumor‐bearing mice, respectively (Table S4, Supporting Information). In comparison, the enhancement extent of GBM on the CE‐T1WI was smaller than the tumor size on the H&E staining slice, which suggested the challenge of identifying infiltrating GBM cells by the contrast‐enhanced MRI technique (Figure S4, Supporting Information). The results demonstrated the superiority of the RS in identifying infiltrating lesions. Moreover, human clinical specimens were collected as the external validation set for further validating the performance of the models. The AUCs of the SVM‐GL261 and SVM‐LN229 models for diagnosing infiltrative tumors were 95.8% and 96.9%, respectively (**Figure** **6**; Table S5, Supporting Information).

To evaluate the GBM cell density threshold accurately detected by RS, we conducted histologic cell counting on 50 infiltrating lesions from human samples. The area of the Raman scan was 0.01 mm^2^ and the cancer cell densities were established. The sample details of the infiltration lesions are displayed in Table S6 (Supporting Information). The detection rate for infiltrating lesions is the main metric for assessing the density threshold because complete detection of the infiltrating lesions to achieve maximal resection is the surgical principle of GBM.^[^ ^3^ ^]^ When the cancer cell densities of infiltrating lesions were ≥1, 2, and 3 cells per 0.01 mm^2^, the detection rates of the SVM‐GL261 and SVM‐LN229 models were 88%, 93.6%, and 100%, respectively. Therefore, 3 cancer cells per 0.01 mm^2^ can be considered as the density threshold of infiltrative tumors identified by RS. An H&E image of the infiltrating lesion with this density is shown in Figure S5 (Supporting Information). The findings demonstrated that the RS can detect micro‐infiltrative lesions at cellular resolution.

## 3 Discussion

In this study, we demonstrated that RS can be used to detect microscopic cancer infiltration in surgical specimens. RS distinguished between GBM infiltrating lesions and normal brain tissues with an accuracy exceeding 95%. Most importantly, the density threshold of infiltrative tumors identified by RS was as low as 3 cancer cells per 0.01 mm^2^ in human specimens. This finding is of great significance for improving the clinical treatment efficacy of GBM because the main objective of GBM surgery is to reduce the volume of residual cancer post‐surgery to prolong survival. RS with cellular resolution enables us to detect micro‐infiltration of tumors, which may achieve complete GBM resection. The distinctive spectral features of infiltrated lesions are the basis for recognition. Most of the spectral intensity of infiltrative lesions was stronger than normal brain tissues, such as most amino acids, lipids, and nucleic acids‐related Raman peaks. The spatial metabolomics analysis based on AFADESI‐MSI was performed to confirm the differences in biochemical metabolites between the infiltrating tumors and normal tissues. The results were consistent with that of Raman analysis, indicating that RS, like metabolomics, provides comprehensive and reliable biological information and is more convenient and cost‐effective. Based on differential spectra, Raman images can reveal the spatial distribution of major biochemical components of tissues. The lesion morphology can be depicted by Raman mapping technology in a label‐free manner, which cannot be achieved with the standard H&E staining methods.

Currently, the commonly used techniques for delineating tumor borders in clinical settings are MRI, PET, and fluorescence imaging technology. Contrast‐enhanced MRI is the preferred imaging modality for both diagnostic evaluation and treatment planning of GBM. However, contrast‐enhanced MRI fails to detect GBM micro‐infiltration lesions. Our results indicated that areas of contrast enhancement do not involve infiltrating cells (Figure S3, Supporting Information), as has been confirmed in previous research.^[^ ^17^ ^]^ Gadolinium‐based contrast agents show contrast enhancement of tumors by crossing the disrupted blood‐brain barrier (BBB) that remains essentially intact in areas of infiltration. Hence, CE‐T1WI is not effective in detecting infiltrative lesions. Several studies have proposed using multimodal MRI, including structural MRI, diffusion tensor imaging (DTI), and magnetic resonance spectroscopy1 (MRS), to evaluate tumor infiltration.^[^ ^18^ ^]^ Li et al. combined CE‐T1WI and DTI to assess tumor infiltration by assessing tumor proliferation.^[^ ^19^ ^]^ Other teams have used DTI combined with MRS to detect peritumoral infiltration areas.^[^ ^20^ ^]^ These studies relied on clinicoradiologic criteria to confirm infiltration lesions and lacked a gold standard in histopathology. Moreover, the diagnostic metrics for infiltrative lesions have not been validated and the diagnostic efficiency has not been reported. In our study, we employed H&E staining as the pathological standard to analyze infiltration lesions. We not only evaluated the performance of the classification model in two types of orthotopic GBMs, but also further validated the ability to identify infiltration lesions in human specimens. The performance of PET is considered to be much better than standard MRI for assessing tumor infiltration.^[^ ^21^ ^]^ Kinoshita and colleagues have demonstrated that ^11^C‐methionine PET technology allows the detection of tumor infiltration lesions with a threshold of 1000 cancer cells per mm^2^.^[^ ^22^ ^]^ However, this resolution is limited to identifying micro‐infiltration. PET imaging is based on the specific uptake of tracer by tumor cells. An accurate diagnosis can only be established when there are a large number of tumor cells and the accumulation of tracer reaches a specific level. The cancer cell threshold for identifying infiltrating lesions by RS is 3 cancer cells per 0.01mm^2^, which enables the detection of micro‐infiltration lesions and facilitates complete tumor removal. Another study that integrated ADC MRI and [^18^F]FET PET to detect glioma infiltration achieved an AUC of 89%.^[^ ^23^ ^]^ In our study, the RS improved the accuracy of identifying infiltrative lesions, with an AUC of up to 95%. In addition, we confirmed the correlation between the spectral intensity of biochemical metabolites, including phospholipids, nucleic acids, UFAs, and proteins, and the density of infiltrative lesions. Robust metrics are provided to objectively quantify the degree of infiltration, which is beneficial for the clinical management of patients. The fluorescence technique has also been explored for delineating tumor boundaries. Based on the 5‐ALA fluorescent agent, margins of tumor tissue fluoresce a light “pink,” reflecting infiltrating disease. However, like PET imaging, the fluorescence technique relies on the uptake of fluorescent dye by tumor cells. Therefore, false negatives often occur in infiltrating areas with low tumor cell density.^[^ ^24^ ^]^ and the diagnostic sensitivity of infiltrating lesions is low.^[^ ^25^ ^]^

The clinical value of these techniques, including MRI, PET, and fluorescence imaging, for elucidating the biological characteristics of GBM is limited. RS can shed light on our understanding of GBM infiltration biology. Specifically, the spectral data from infiltrative lesions and normal brain tissues revealed significant differences in FAs, nucleic acids, phospholipids, proteins, and amino acids content. A higher FAs content was observed in infiltrating tumors. The upregulation of FAs synthesis is a classically described metabolic alteration in cancer. Several studies have confirmed the important roles of FA metabolism in tumor growth and metastasis.^[^ ^26^ ^]^ It has been reported that increased expression of FA synthase (FASN) in GBM supports cytosolic acetyl‐CoA levels, which can be used for lipogenesis.^[^ ^27^ ^]^ Moreover, cancer cells that are abundant in UFAs tend to display fluid membranes due to the high fluidity of UFAs, which contributes to migration and facilitates cancer infiltration.^[^ ^28^ ^]^ Similarly, the infiltrating lesions contain more nucleic acids than normal tissues. It is well known that the high proliferation rate, a hallmark of cancer, requires large amounts of nucleic acids, which is critical in promoting tumor cell survival and proliferation.^[^ ^29^ ^]^ In addition, Zhou and coworkers observed that purine metabolism regulates DNA repair, which leads to treatment resistance in GBM.^[^ ^30^ ^]^ Conversely, the content of phospholipids is significantly decreased in infiltrative tumors, because infiltrative tumors destroy the normal structure of the myelin sheath mainly composed of phospholipids. Additionally, it has been shown that glioma cells have markedly lower levels of phospholipids compared to non‐tumor cells, and low phospholipid levels are associated with tumorigenicity.^[^ ^31^ ^]^ Dysregulation of protein metabolism is considered a primary metabolic marker of tumor cells. Infiltrative tumors display high protein levels, which is in agreement with emerging evidence suggesting increased energy and biomass generation to support tumor growth.^[^ ^32^ ^]^ and infiltration.^[^ ^33^ ^]^ Remarkably, we observed reduced tryptophan levels in infiltrative GBM. It was previously discovered that the upregulation of tryptophan metabolism promotes tumor infiltration and progression. The tryptophan metabolite, Kyn, activates AhR via the Kyn‐AhR‐aqp4 signaling pathway to promote cell motility.^[^ ^34^ ^]^ In summary, RS can clarify the biological behavior of GBM infiltration by alterations in biochemical metabolites.

Notably, our study employed a benchtop Raman instrument to evaluate GBM micro‐infiltrating lesions in vitro. Our future research will focus on employing a handheld Raman spectrometer for GBM study in vivo and establishing a standardized Raman diagnostic process, which is essential for intraoperative application in neurosurgery. Another weakness of RS is its limited field of detection. Combining RS with fluorescence imaging.^[^ ^35^ ^]^ can enhance its effectiveness. Fluorescence imaging could indicate the possible locations of GBM infiltration and further confirm the presence of infiltrating cells by RS.^[^ ^36^ ^]^

In conclusion, RS identifies micro‐infiltration lesions with superior resolution, which surpasses traditional methods. The findings show the tremendous clinical significance of RS as a powerful tool for precisely identifying GBM micro‐infiltration. This technique will ultimately assist in the safe and precise resection of GBM, and thus improve patient survival.

## 4 Experimental Section

### Ethics and Patient Samples

To validate the power of the classification model, a dataset from 8 patients with pathology‐proven GBM was included. All procedures involving patient materials were conducted according to the guidelines approved by the Ethics Committee of the General Hospital of Eastern Theater Command (2021DZGZR‐YBB‐066). The informed consent of all participating subjects was obtained. Tumor and peritumoral tissue samples from patients were evaluated by two pathologists.

### Cell Culture

The human GBM cell lines, LN229 and U87 MG, were purchased from the American Type Culture Collection (Manassas, VA, USA). The murine GBM cell line, GL261, was purchased from Cellverse Bioscience Technology Co., Ltd (Shanghai, China). The cell lines were authenticated by short tandem repeat analysis and tested for Mycoplasma (Myco‐lumi luminescent mycoplasma detection kit, KeyGEN BioTECH). Cells were cultured in DMEM (Gibco, 8123167) containing 10% fetal bovine serum (Gibco, 2547014P) and 1% penicillin‐streptomycin (Gibco, 10378016). The cells were incubated in a 5% CO_2_ humidified incubator at 37 °C.

### Orthotopic GBM Models

Female C57BL/6 (18‐20 g) and BALB/c nude mice (17‐19 g) (Huachuang Sino, China), 5–7 weeks old, were housed under standard conditions. To establish the orthotopic GBM models, 1 × 10^6^ cells suspended in 8 µL of phosphate‐buffered saline were implanted into the striatum. GL261 cells were injected intracranially into 16 C57BL/6 mice, and LN229 and U87 MG cells were injected intracranially into 16 and 3 BALB/c nude mice, respectively. The implantation site was 2 mm lateral and 1 mm anterior to the bregma, and the cells were implanted 3.5 mm deep into the brain surfaces using a stereotactic fixation device (RWD, China). Tumors were confirmed using MRI (BioSpec 94/30 USR; Bruker, Germany) after 3–4 weeks. Animal experiments were conducted according to the guidelines approved by the Ethics Committee of the General Hospital of Eastern Theater Command (2021DZGKJDWLS‐0029).

### Conventional MR Imaging

T1‐weighted imaging was performed using a small animal MR scanner with a mouse head volume coil (BioSpec 94/30 USR; Bruker, Germany). T1‐weighted images (TR/TE, 800/9; field of view, 18 × 18 mm; matrix, 512 × 512; section thickness, 0.7 mm; and number of average, 10) were acquired after the injection of a gadolinium‐based contrast agent (0.2 mL kg^−1^ of gadobutrol [Gadovist]; Beilu Pharmaceutical Co., Ltd, Beijing, China). The enhancement region on the CE‐T1WI was delineated as the region of interest by a senior radiologist. The area of enhancement was calculated using RadiAnt DICOM Viewer.

### Brain Slice Sample Preparation

Tumor‐bearing mice were deeply anesthetized with an intraperitoneal injection of sodium thiopental and perfused with saline in succession via the heart to douche blood before decapitation, followed by brain extraction. Sixteen tissue samples with paired cancer cores and peritumoral tissues were obtained from 8 patients diagnosed with GBM. The tumor‐bearing mouse brains and surgically resected human tissue samples were snap‐frozen in liquid nitrogen and stored at −80 °C. The tissue sections (5‐µm thick) that were prepared by a microtome cryostat were attached to the stainless steel substrate for Raman measurements. Adjacent 5‐µm thick tissue sections were used for H&E staining.

### Raman Measurements and Analysis

The Raman spectra of tissue samples were collected by an inVia confocal Raman microscope (Renishaw, UK) with a 785 nm wavelength. An L×50 objective (numerical aperture [NA] = 0.50, working distance [WD] = 8.2 mm) focused the laser beam onto the surface of tissue slices. Each spectrum was integrated for 10 s (10 accumulations of 1 s each) at 100 mW laser power. A site refers to a Raman scan area of 100 × 100 µm. The spectra of each site were averaged to generate a representative spectrum. The spectra data were processed using WIRE 4.3 software with cosmic ray removal, Savitzky–Golay smoothing, baseline correction, and normalization. The Raman mapping step size was 2 µm and the mapping range was adjusted according to the lesion size. Raman mapping analysis was conducted using WiRE 4.3 software.

### Machine Learning

An SVM was used to construct the Raman models on the Deepwise Multimodal Research Platform version 2.3 (https://keyan.deepwise.com, Beijing Deepwise & League of PHD Technology Co., Ltd, China). A Pearson feature correlation analysis was performed, and those with a correlation coefficient ≥0.7 were eliminated. The ANOVA test was also performed for further feature selection. The samples were split into training and test sets at a 3:1 ratio. Spectral data from 96 sites in randomly selected GL261 tumor‐bearing mice were used to build a classification model, namely SVM‐GL261. Spectral data from 32 sites of GL261 tumor‐bearing mice that underwent CE‐T1WI were used for testing. Spectral data from 32 sites of human tissue samples were used as the external validation set. The same split of the dataset was performed in the LN229 tumor‐bearing mice and an SVM‐LN229 classification model was built. Spectral data from 90 sites of human tissue samples were used as the external validation set. Indicators for evaluating SVM models include accuracy, AUC, sensitivity, and specificity. Furthermore, the detection rate of SVM models is assessed for GBM infiltrative lesions with different tumor cell densities. The density threshold for identifying infiltrative tumors by RS is evaluated based on the detection rate.

### Neuropathology Assessment

The pathology analyses were conducted independently by two neuropathologists. In H&E images, atypical cells were identified by assessing the morphological features, including nuclear atypia and nuclear polymorphism. The extent of GBM on the H&E image, including the infiltrative area and solid tumor, was delineated and the size was calculated using an Olympus OLyVIA 3.3.

### Spatial Metabolomics Analysis

A tumor‐bearing tissue slice was subjected to mass spectrometry imaging analysis. Region‐specific MS profiles were extracted by matching H&E images with the spatial shrunken centroids clustering result. Detailed experimental methods are described in Supplemental Text (Supporting Information).

### Statistical Analysis

All statistical analyses were conducted in GraphPad Prism 9.0 software. All data are presented as means ± standard deviation (SD) as well as medians and interquartile ranges. Peak intensity comparisons between normal tissues and infiltrative lesions were conducted using two‐tailed unpaired t‐tests or Mann‐Whitney test, as applicable. The correlation between the two factors was assessed by Pearson correlation analysis. A *P* < 0.05 was considered statistically significant. *: *P* < 0.05, **: *P* < 0.01, ***: *P* < 0.001.

## Conflict of Interest

The authors declare no conflict of interest.

## Supporting information

Supporting Information

## Data Availability

The data that support the findings of this study are available from the corresponding author upon reasonable request.
