# Supplementary material for: Precise Identification of Glioblastoma Micro‐Infiltration at Cellular Resolution by Raman Spectroscopy
Source: Adv Sci (Weinh). 2024 Jul 31;11(36):2401014. doi: 10.1002/advs.202401014 (PMC11423152; doi:10.1002/advs.202401014)
Supplement: Supplementary file 1 — Supporting Information [file ADVS-11-2401014-s001.docx]

**Supporting Information**

**Precise Identification of Glioblastoma Micro-Infiltration at Cellular Resolution by Raman Spectroscopy**

*Lijun Zhu, Jianrui Li, Jing Pan, Nan Wu, Qing Xu, Qing-Qing Zhou, Qiang Wang, Dong Han, Ziyang Wang, Qiang Xu, Xiaoxue Liu, Jingxing Guo, Jiandong Wang, Zhiqiang Zhang, Yiqing Wang, Huiming Cai, Yingjia Li, Hao Pan,* Longjiang Zhang,* Xiaoyuan Chen,* Guangming Lu**


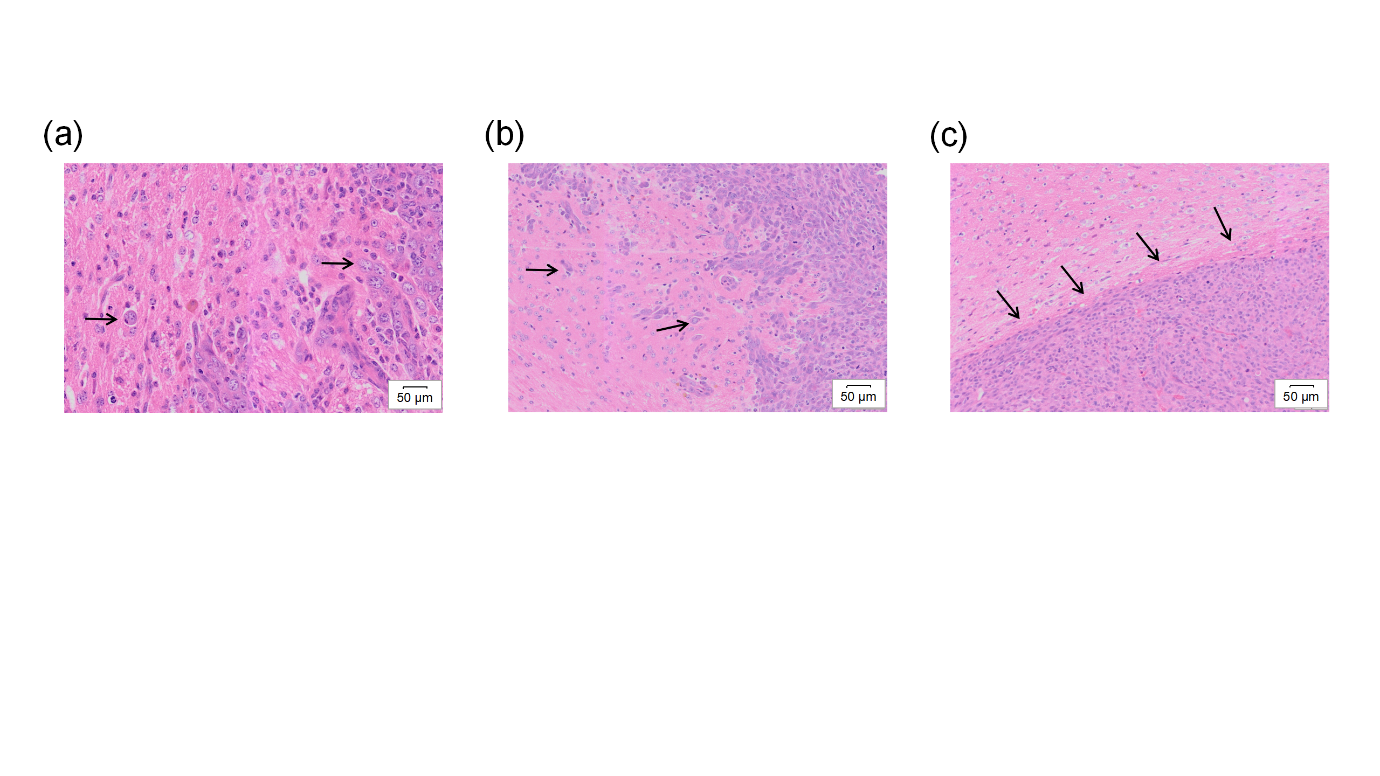


**Figure S1.** GBM cell lines exhibit different growth patterns. The GBMs formed by GL261 (a, n = 3) and LN229 cells (b, n = 3) display significant infiltration. The arrows point at invading tumor lesions. c) The GBM formed by U87 MG cells (n = 3) shows a circumscribed growth pattern and the arrows point to the tumor border.

**Table S1.** The Raman peak positions and major assignments of the normal tissues and infiltrative tumors.

| Position (cm^-1^) | Major assignment |
| --- | --- |
| 622  643  667  702 | Phenylalanine  Proline  Cystine  Cholesterol |
| 716  744  758 | Phospholipid, Choline  Tryptophan  Tryptophan |
| 780  828 | Nucleic acid  Glutathione |
| 852  928  959 | Glycogen  Cystine, Collagen  Proline |
| 1003  1102  1125 | Phenylalanine  Glycogen  Protein |
| Position (cm^-1^) | Major assignment |
| 1156 | Carotenoid, Protein |
| 1174  1206 | Saturated fatty acid  Tyrosine, Phenylalanine |
| 1264  1305 | Unsaturated fatty acid  Triglyceride |
| 1448 | Lipid, Glutathione |
| 1572 | Nucleic acid |
| 1586 | Phenylalanine |
| 1606 | Tyrosine |
| 1616  1658 | Tyrosine, Tryptophan  Lipid, Protein |


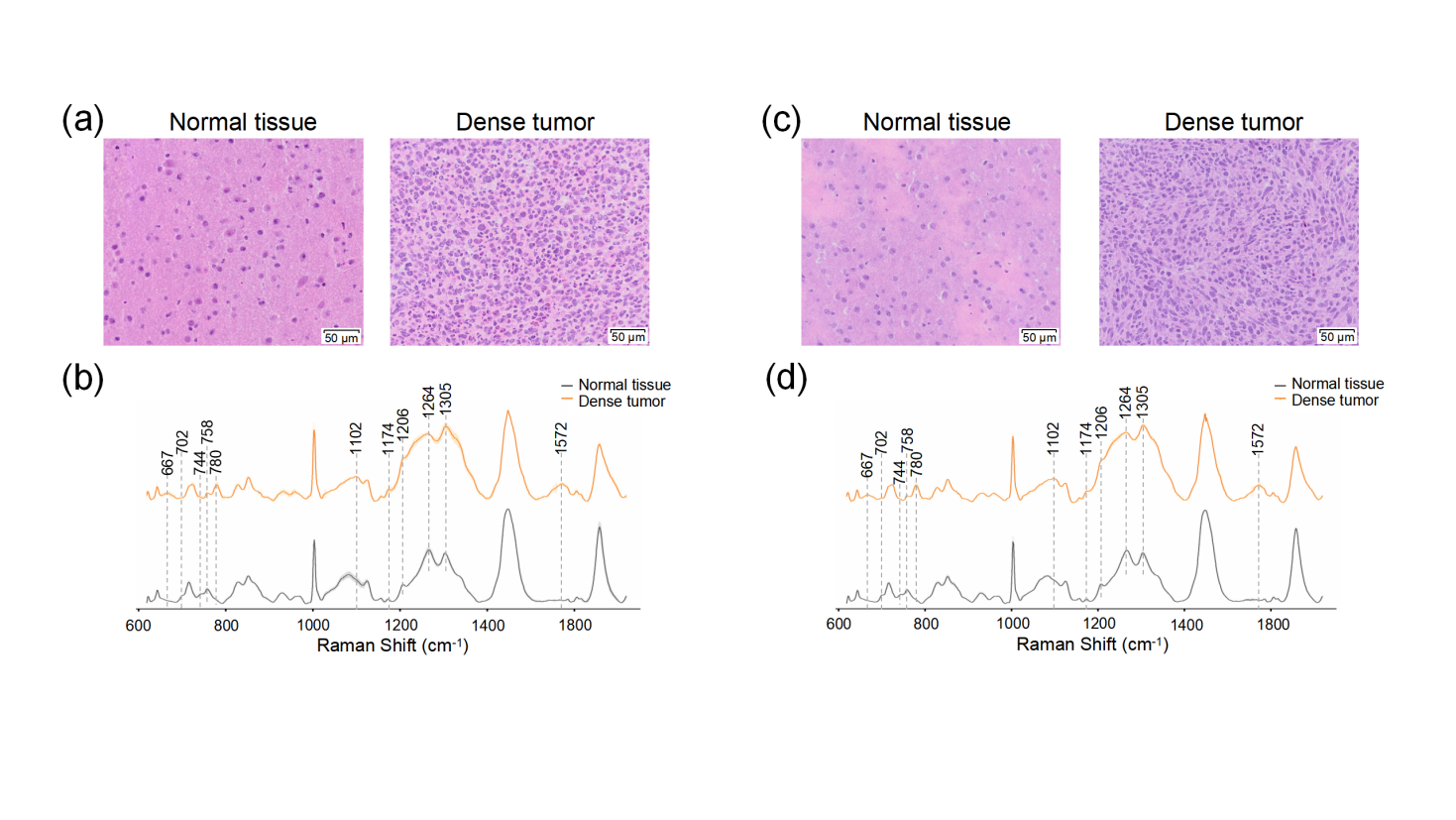


**Figure S2.** Comparison of normal brain tissues and dense tumors in (a,b) GL261 and (c,d) LN229 tumor-bearing mice (n = 16 per animal model). a,c) The H&E images of normal brain tissues and dense tumors. b,d) The average Raman spectra of normal brain tissues and dense tumors (n = 64 per group). The shaded areas represent the standard deviations.


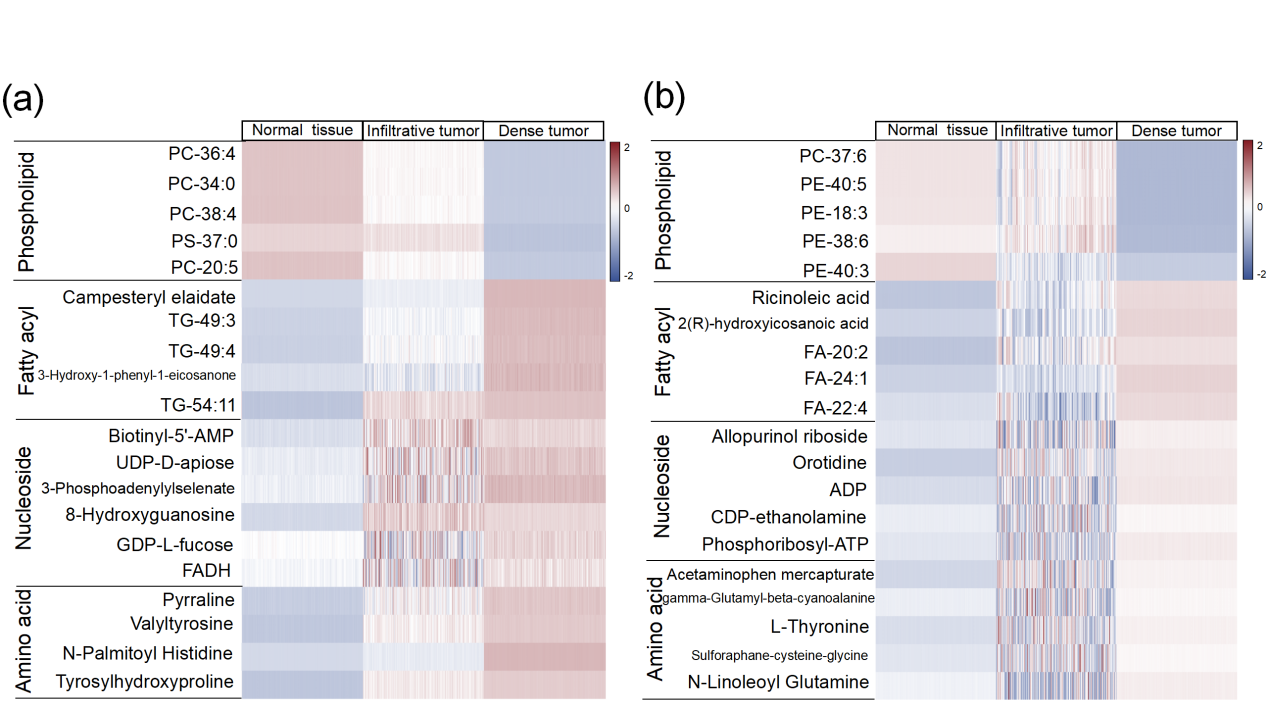


**Figure S3.** Heat maps of AFADESI-MSI data in ESI+ (a) and ESI− modes (b) showing various classes of metabolites for normal tissues (the 1^st^, 2^nd^, 6^th^ and 7^th^ clusters), infiltrative tumors (the 9^th^ cluster) and tumor masses (The 4^th^ and 12^th^ clusters). Abbreviations: PC (phosphatidylcholine), PE (phosphatidylethanolamine), PS (phosphatidylserine), TG (triglyceride), and FA (fatty acid).

**Table S2.** The capability of RS to identify dense tumors in human samples.

| Model | Accuracy  (%) | AUC  (%) | Sensitivity  (%) | Specificity  (%) |
| --- | --- | --- | --- | --- |
| SVM-GL261 | 96.9 | 100 | 93.8 | 100 |
| SVM-LN229 | 100 | 100 | 100 | 100 |

**Table S3.** Modeling features of SVM.

| Raman features (cm^-1^) | | Raman band assignment |
| --- | --- | --- |
| SVM-GL261 | SVM-LN229 |  |
| 643 | 643 | Proline |
|  | 702 | Cholesterol |
| 716 | 716 | Phospholipid, Choline |
| 758 | 758 | Tryptophan |
|  | 780 | Nucleic acid |
| 921 | 921 |  |
| 982 |  |  |
| 1003 | 1003 | Phenylalanine |
| 1033 |  |  |
| 1107 |  |  |
|  | 1438 |  |
| 1486 |  |  |
|  | 1694 |  |
| 1719 | 1719 |  |

**Table S4.** The capability of RS combined with SVM to identify GBM infiltrative lesions in tumor-bearing mice.

| Model | Accuracy  (%) | AUC  (%) | Sensitivity  (%) | Specificity  (%) |
| --- | --- | --- | --- | --- |
| SVM-GL261 | 90.6 | 98.4 | 87.5 | 93.8 |
| SVM-LN229 | 93.8 | 96.1 | 87.5 | 100 |


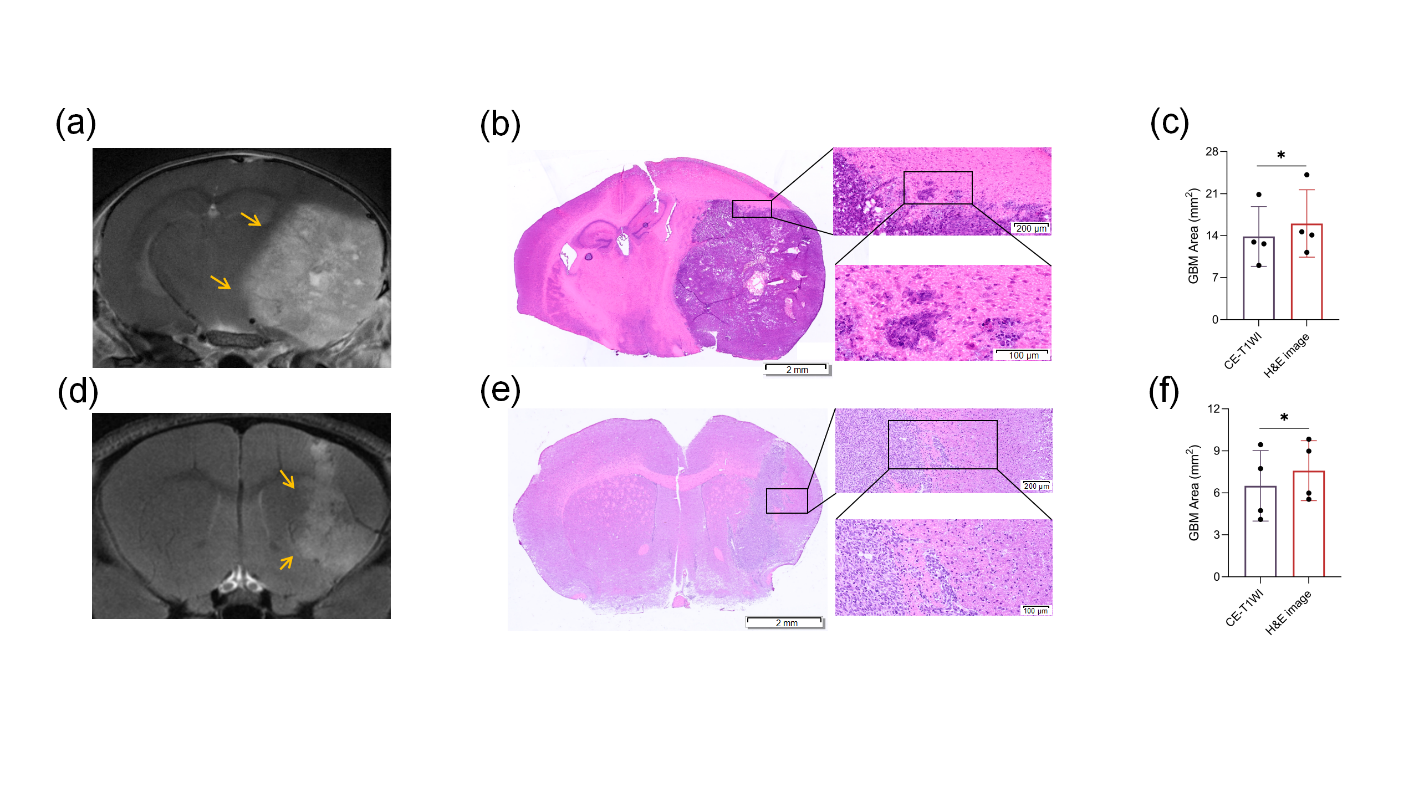


**Figure S4.** The comparison of tumor sizes in the CE-T1WI and H&E image for GL261 (a-c, n = 4) and LN229 GBM (d-f, n = 4). Enhancement extent of GBM was 20.85 mm^2^ (a) and 4.73 mm^2^ (d). Masses (arrows) exhibit diffuse enhancement. The H&E images of GBM at the corresponding CE-T1WI section. Tumor sizes were 24.13 mm^2^ (b) and 5.99 mm^2^ (e). The black boxes are the infiltrative areas. c,f) Enhancement extent of tumors in the CE-T1WI and tumor sizes in H&E images.

**Table S5.** The capability of RS combined with SVM to identify GBM infiltration lesions in human specimens.

| Model | Accuracy  (%) | Sensitivity  (%) | Specificity  (%) |
| --- | --- | --- | --- |
| SVM-GL261 | 92.2% | 88% | 97.5% |
| SVM-LN229 | 92.2% | 88% | 97.5% |

**Table S6**. Sample sizes of GBM infiltrating lesions with different tumor cell densities.

| Sample sizes | Cancer cell density  (cells/0.01 mm^2^) |
| --- | --- |
| 2 | 18 |
| 2 | 17 |
| 4 | 16 |
| 3 | 15 |
| 1 | 14 |
| 1 | 13 |
| 1 | 12 |
| 2 | 11 |
| 3 | 10 |
| 2 | 9 |
| 4 | 8 |
| 3 | 7 |
| 4 | 6 |
| 5 | 5 |
| 4 | 4 |
| 3 | 3 |
| 3 | 2 |
| 3 | 1 |

**
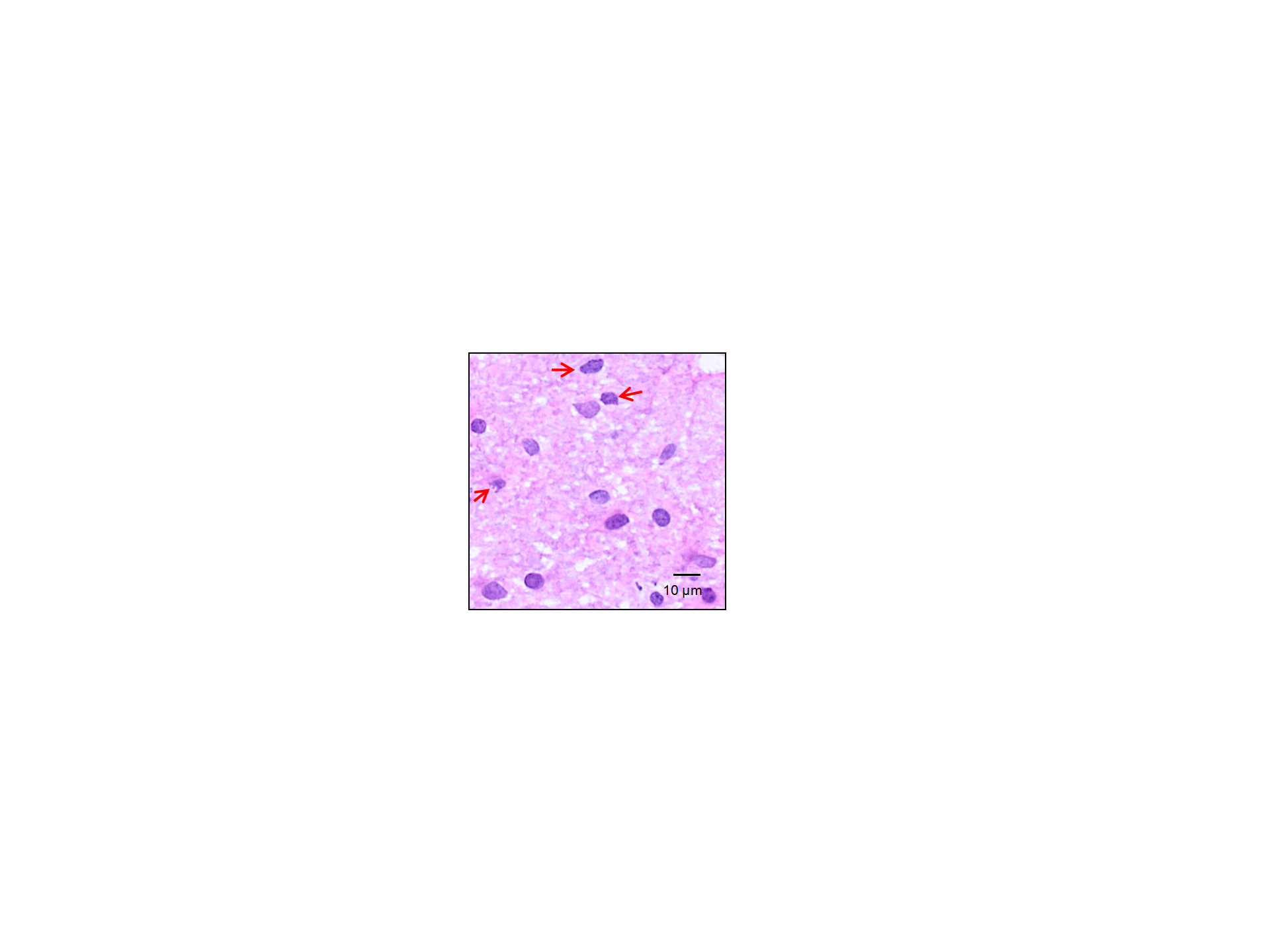
**

**Figure S5.** An H&E staining image of the infiltrating lesion with a density of 3 cancer cells/0.01 mm^2^. In the infiltrating lesion, the red arrows are the infiltrative cancer cells and the others are normal cells.

Supplemental Text

Data of AFADESI-MSI

Traditional MSI is not suitable for the analysis of invasive lesions due to the limited invasion volume for sampling. AFADESI-MSI-mediated spatial metabolomics enabled us to simultaneously detect hundreds of metabolites in situ for each voxel and take into account the spatial variation of metabolites. The spatial resolution applied in this study was set to 20 μm. In the positive electrospray ionization source (ESI+) mode, 760 ions, and in the negative electrospray ionization source (ESI-) mode, 560 ions were identified and selected as candidate differential metabolites.

Metabolic profile of the tumor masses

The metabolism of tumor masses (the 4^th^ and 12^th^ clusters) is characterized by four main categories: phospholipids, fatty acyls, nucleosides, and amino acids. Compared with normal brain tissues (the 1^st^, 2^nd^, 6^th^ and 7^th^ clusters) and infiltrative tumors (the 9^th^ cluster), tumor bulks contained higher levels of fatty acyls, nucleosides and amino acids, and lower levels of phospholipids, which is consistent with previous findings.^[1]^

Experimental Section

Spatial metabolomics analysis: a 10 μm tumor-bearing tissue slice was subjected to mass spectrometry imaging analysis and an adjacent slice was used for H&E staining. An air AFADESI-MSI platform (Beijing Victor Technology Co., LTD, China) was utilized. The MS mass range was 70-1000 Da. The AFADESI-MSI experiment was carried out with a 20 μm vertical step in the y direction. Region-specific MS profiles were accurately extracted by matching high-spatial resolution H&E images with the spatial shrunken centroids clustering result. Differential metabolites were chosen based on variable importance of projection (VIP) values > 1.0 and *P* < 0.05. The ions detected by AFADESI were annotated using the pySM pipeline and an in-house SmetDB database (Lumingbio, China).^[2]^

References

[1] S. Murugappan, S. A. M. Tofail, N. D. Thorat, *ACS Omega* 2023, 8, 27845.

[2] Andrew Palmer, Prasad Phapale, Ilya Chernyavsky, Regis Lavigne, Dominik Fay, Artem Tarasov, Vitaly Kovalev, Jens Fuchser, Sergey Nikolenko, Charles Pineau, Michael Becker, Theodore Alexandrov, *Nat Methods*. 2017, 14, 1.
